# Supplementary material for: Performance of the Vitek 2 Advanced Expert System (AES) as a Rapid Tool for Reporting Antimicrobial Susceptibility Testing (AST) in Enterobacterales from North and Latin America
Source: Microbiol Spectr. 2023 Jan 16;11(1):e04673-22. doi: 10.1128/spectrum.04673-22 (PMC9927136; doi:10.1128/spectrum.04673-22)
Supplement: Supplemental file 1 — Tables S1 and S2. Download spectrum.04673-22-s0001.pdf, PDF file, 0.1 MB [file spectrum.04673-22-s0001.pdf]

Supplemental Material

Table S1. Distribution of *Enterobacterales* isolates by species and organism source

***Enterobacterales* species / group**	**SENTRY Surveillance Program**	**CDC AR Bank**
Citrobacter freundii	1	2
Citrobacter freundii species complex	19	
Citrobacter koseri	11	2
Enterobacter cloacae	0	9
Enterobacter cloacae species complex	61	2
Escherichia coli	111	23
Klebsiella aerogenes	6	2
Klebsiella oxytoca	16	2
Klebsiella pneumoniae	130	47
Kluyvera ascorbata	0	1
Morganella morganii	3	2
Pluralibacter gergoviae	1	
Proteus mirabilis	14	4
Providencia rettgeri	4	1
Providencia stuartii	3	
Serratia marcescens	27	9
**Total**	**407**	**106**

Table S2. Distribution of *Enterobacterales* isolates by geographic region

Continent/country	no. of isolates
**Latin America**	**123**
Argentina	34
Brazil	33
Chile	24
Guatemala	1
Mexico	29
Venezuela	2
**North America**	**284**
USA	284
**Unknown**	**106**
CDC bank	106
**Total**	**513**
